# Urinary Benzene Biomarkers and DNA Methylation in Bulgarian Petrochemical Workers: Study Findings and Comparison of Linear and Beta Regression Models

**DOI:** 10.1371/journal.pone.0050471

**Published:** 2012-12-05

**Authors:** Wei Jie Seow, Angela Cecilia Pesatori, Emmanuel Dimont, Peter B. Farmer, Benedetta Albetti, Adrienne S. Ettinger, Valentina Bollati, Claudia Bolognesi, Paola Roggieri, Teodor I. Panev, Tzveta Georgieva, Domenico Franco Merlo, Pier Alberto Bertazzi, Andrea A. Baccarelli

**Affiliations:** 1 Department of Environmental Health, Harvard School of Public Health, Boston, Massachusetts, United States of America; 2 Department of Clinical Sciences and Community Health, University of Milan & Istituto Di Ricovero e Cura a Carattere Scientifico Fondazione Ca Granda Maggiore Policlinico Hospital, Milan, Italy; 3 Department of Biostatistics, Harvard School of Public Health, Boston, Massachusetts, United States of America; 4 Department of Cancer Studies and Molecular Medicine, University of Leicester, Leicester, United Kingdom; 5 Department of Chronic Disease Epidemiology, Yale School of Public Health, New Haven, Connecticut, United States of America; 6 Environmental Carcinogenesis Unit, Istituto Di Ricovero e Cura a Carattere Scientifico AOU San Martino-IST-National Cancer Research Institute, Genoa, Italy; 7 Department of Chemical Analyses, National Centre of Public Health and Analyses, Sofia, Bulgaria; 8 Department of Public Health and Risk Assessment, National Center of Public Health and Analyses, Sofia, Bulgaria; 9 Epidemiology, Biostatistics and Clinical Trials, Istituto Di Ricovero e Cura a Carattere Scientifico AOU San Martino-IST-National Cancer Research Institute, Genoa, Italy; Cleveland Clinic Lerner Research Institute, United States of America

## Abstract

Chronic occupational exposure to benzene is associated with an increased risk of hematological malignancies such as acute myeloid leukemia (AML), but the underlying mechanisms are still unclear. The main objective of this study was to investigate the association between benzene exposure and DNA methylation, both in repeated elements and candidate genes, in a population of 158 Bulgarian petrochemical workers and 50 unexposed office workers. Exposure assessment included personal monitoring of airborne benzene at work and urinary biomarkers of benzene metabolism (S-phenylmercapturic acid [SPMA] and trans,trans-muconic acid [t,t-MA]) at the end of the work-shift. The median levels of airborne benzene, SPMA and t,t-MA in workers were 0.46 ppm, 15.5 µg/L and 711 µg/L respectively, and exposure levels were significantly lower in the controls. Repeated-element DNA methylation was measured in Alu and LINE-1, and gene-specific methylation in *MAGE* and *p15*. DNA methylation levels were not significantly different between exposed workers and controls (*P*>0.05). Both ordinary least squares (OLS) and beta-regression models were used to estimate benzene-methylation associations. Beta-regression showed better model specification, as reflected in improved coefficient of determination (pseudo R^2^) and Akaike’s information criterion (AIC). In beta-regression, we found statistically significant reductions in LINE-1 (−0.15%, *P*<0.01) and *p15* (−0.096%, *P*<0.01) mean methylation levels with each interquartile range (IQR) increase in SPMA. This study showed statistically significant but weak associations of LINE-1 and *p15* hypomethylation with SPMA in Bulgarian petrochemical workers. We showed that beta-regression is more appropriate than OLS regression for fitting methylation data.

## Introduction

Benzene is a known human carcinogen classified by the International Agency for Research on Cancer (IARC) as a Group I carcinogen due to sufficient evidence for causing acute myeloid leukemia (AML) in humans [1]. It has also been associated with other diseases of the blood-forming systems such as myelodysplastic syndromes (MDS), lymphocytic leukemias and other cancers [2]–[5]. Some of the major current exposures to benzene occur in petrochemical industries, coke and steel plants, and from traffic exhaust and cigarettes smoke. Following findings on the effects of low-dose occupational exposure to benzene, such as hematotoxic effects seen at levels as low as 1 ppm [6], whether there is a safe level of occupational benzene exposure has been highly debated. Once absorbed into the body, benzene is metabolized to benzene oxide before forming either S-phenylmercapturic acid (SPMA) or trans,trans-muconic acid (t,t-MA) in the liver and lungs [7]. SPMA and t,t-MA are the most commonly used biomarkers of occupational benzene exposure [8]–[10]. The exact mechanism by which benzene exerts its toxicity still remains unclear.

Aberrant DNA methylation, which may lead to genomic instability and altered gene expression, is frequently observed in AML [11]. Repeated-element DNA hypomethylation, as well as gene-specific hypermethylation or hypomethylation are commonly seen in hematological cancers [12]. Hypermethylation of tumor suppressor *p15*, which leads to inactivation of the gene and subsequent uncontrolled proliferation of the cell, is commonly observed in AML and other hematological cancers [13]–[16]. The melanoma antigen family A (*MAGE)* gene, which encodes tumor-rejection antigens, is widely expressed in cells from hematological malignancies and is found to be hypomethylated with benzene exposure [17]–[20]. Studies have found significant correlation between SPMA and 8-oxo-2′-deoxyguanosine (8-oxodG) – a biomarker of oxidative DNA damage – measured in lymphocytes, which may then induce epigenetic modifications [21], [22].

To date, most studies have performed statistical analyses of DNA methylation using ordinary least squares (OLS) regression, in which the Gaussian distribution is assumed for the methylation level. However, methylation is usually measured as percentage or proportion values (%5 mC), which are bounded in the (0,1) interval. In principle, the beta distribution, as opposed to the Gaussian distribution, would be the most natural statistical model for analyzing proportional data bounded in the (0,1) interval [23] and has also been shown in many empirical studies, to be an appropriate distributional model for proportion data where most of the linear assumptions tend to fail [24]. Beta regression has been proven to be more flexible than OLS regression, as it uses two sets of parameters to model trends in both the conditional mean and the precision (hence, conditional variance) of the proportion data distributed over the (0,1) interval with respect to a set of explanatory covariates [25]. However, whether a beta distribution is more appropriate to fit methylation data has yet not been evaluated on empirical data.

The main objective of this study was to investigate possible mechanisms of benzene toxicity via DNA methylation, by examining both repeated-element and gene-specific DNA methylation in a population of Bulgarian petrochemical workers. We also explored using beta-regression models to fit the methylation data as a methodological alternative to more commonly used OLS regression. Since DNA methylation is potentially modifiable through lifestyle or pharmacological intervention, identifying the association between benzene and methylation may be critical to identify biological effects from benzene exposure and design public health interventions among exposed workers.

## Materials and Methods

### Ethics Statement

Written informed consent was obtained prior to enrollment. The study was approved by the Genoa, Italy National Cancer Research Institute Ethics Committees.

### Study Population

The study population consisted of 158 petrochemical workers employed at the Lukoil-Neftochim petrochemical plant in Burgas, Bulgaria and 50 unexposed office workers, used as controls, from the same plant. All subjects were recruited between October 1999 and September 2000 and had worked in the same position at the plant for at least one year. Self-administered questionnaires collected information regarding exposure history, medical history, lifestyle factors, smoking habits, occupational activities and other personal information.

### Benzene Exposure Assessment

Individual airborne benzene measurements were based on personal monitoring sampling on the day before phlebotomy. Personal exposure to airborne benzene was measured by active sampling using charcoal sampling tubes as described previously [26]. Briefly, personal full-shift air monitoring was performed during one working day using active samplers with flow of 20–30 cm^3^/min worn near the breathing zone during a typical work-shift (midweek, from 8∶00 a.m. to 2∶00 p.m.), and analyzed with a Perkin Elmer Model 8300 gas chromatograph with flame-ionization detector and column DB-1–30 m. The limit of detection (LOD) for benzene was 0.023 ppm for the whole sample volume.

In addition to air benzene, urinary biomarkers of benzene exposure, i.e., S-phenylmercapturic acid (SPMA) and trans-trans-muconic acid (t,t-MA), which represent intermediary metabolites of benzene metabolism, were also assessed. Both biomarkers were measured in urine samples collected at the end of the work shift in the same day of air monitoring using previously-described methods [26], [27]. Briefly, determination of urinary t,t-MA was performed by pre-purification of urine with solid-phase extraction using a strong-anion exchange column (SAX, 300 mg, Supelco Analytical, St. Louis, MO) followed by high performance liquid chromatography and UV detection as reported by Buratti and colleagues [28]. The LOD for t,t-MA was 50 µg/l, and forty-six measurements below LOD were assigned a value corresponding to half of LOD (i.e. 25 µg/l) with thirty-three (71.7%) of them being controls. Determination of urinary SPMA was based on an immunoassay technique according to a previously published procedure [29]. The immunoassay plate-based kit and reagents were from AB Biomonitoring Ltd. (Cardiff, Wales, United Kingdom). The detection limit of the procedure was 0.2 µg/L and none of the measurements were below LOD.

### Methylation Outcomes

Four ml of whole blood was collected in EDTA tubes from each subject. DNA was extracted using the Wizard Genomic DNA purification kit (Promega, Madison, WI, USA) following the manufacturer’s instructions. We performed DNA methylation analyses on bisulfite-treated DNA using a highly-quantitative analysis based on PCR-Pyrosequencing. 500 ng DNA (concentration 25 ng/µl) was treated using the EZ DNA Methylation-Gold™ Kit (Zymo Research, Orange, CA, USA) according to the manufacturer’s protocol. Final elution was performed with 200 µl M-Elution Buffer. We performed DNA methylation analyses of Alu and LINE-1 repeated sequences, which allow for the amplification of a representa­tive pool of repetitive elements, as previously described [17].

To measure methylation of the *MAGE* and *p15* promoters, a 50 µL PCR was carried out in 25 µL GoTaq Green Master mix (Promega, Madison, WI, USA), 25 ng bisulfite-treated genomic DNA, with primer concentrations and PCR cycling conditions for each assay as shown in Table S1. PCR products were purified and sequenced by pyrosequencing as previously described using 0.3 µΜ sequencing primer [17]. PCR primers are shown in Table S2. Compared to other common methods of DNA methylation analysis, pyrosequencing-based assays have the advantage of producing individual measures of methylation at more than one CpG dinucleotide, thus more accurately reflecting DNA methylation in the region. In each gene promoter assay, we measured %5 mC at different CpG dinucleotides within the promoter region as shown in Table S3. Every sample was pyrosequenced two times for each assay to confirm reliability and the average of the two runs was used in the statistical analysis.

### Statistical Analysis

Demographic, exposure and methylation variables were compared between workers and controls using Pearson’s chi-square test for categorical variables, Welch two-sample t-test for normally-distributed continuous variables and Wilcoxon’s rank sum test for non-normally distributed continuous variables. First, simple linear ordinary least squares (OLS) models were fitted to Alu, LINE-1, *MAGE,* and *p15* methylation levels as dependent variables with each of the exposure variables separately. Additional multiple linear regression models were fitted that adjusted for the set of potential confounders of age, sex, current smoking habits, daily hours of environmental tobacco smoke (ETS) exposure and education level. Since DNA methylation was measured in white blood cells, and each white blood cell subtype may have different methylation profiles [30], we also measured proportions of the various leukocyte cell types (basophils, eosinophils, lymphocytes, monocytes and neutrophils) and adjusted for all analyses in order to eliminate influences from differences in proportions of cell types (Table S4). We then fit a second set of models using beta-regression [31]. We hypothesized that the beta-regression approach to be more appropriate and potentially provide more statistical power than OLS regression for methylation data, which are measured as proportions ranging from 0 to 1 (reported here, as customary, as percent values). Both the conditional mean and the precision (hence, conditional variance) of each methylation variable were modeled with the same set of covariates using the logit link for the conditional mean response and the log link for the conditional precision. The estimates from all models are reported as ‘increase in percent methylation per IQR increase in exposure’ in order to make the associations in the different models comparable for all exposure variables. Fitted mean methylation level trajectories for an individual with average characteristics (40 years old, non-smoking male worker with middle-school education and 5.3 ETS [Environmental Tobacco Smoking] hours) were plotted to provide a graphical representation of the results. Regular adjusted (for OLS) and pseudo adjusted (for beta regression) coefficients of determination (R^2^), as well as Akaike’s information criterion (AIC) were also computed for each of the adjusted models in order to compare relative goodness of fit between OLS and beta regression models. We also generated Pearson residual plots to compare model fit (Figures S1 and S2). All statistical analyses were conducted using the *betareg* and base packages in R version 2.13.1 (R-project.org. © 1998–2012) All tests were conducted as two-sided and considered significant with a p-value <0.05.

## Results

The study participants were predominantly male and most of the individual characteristics were similar in both workers and controls (Table 1). However, education level was significantly lower in workers as compared to controls (*P*<0.001). ETS hours were significantly higher in workers compared to controls (*P*<0.001). Therefore both education and ETS hours were adjusted for in our regression models.

**Table 1:** Characteristics of 158 Petrochemical Workers and 50 Controls, Bulgaria from 1999– 2000.

Variables^a^		Workers	Controls	*P*-value^b^
Sex	Male	138 (87.3%)	38 (76%)	0.089
	Female	20 (12.7%)	12 (24%)	
Age, years		39.9 (8.39)	41.3 (10.8)	0.41
Education level	None/Elementary School	13 (8.23%)	2 (4%)	<0.001*
	Middle School	121 (76.6%)	21 (42%)	
	High School and above	24 (15.2%)	27 (54%)	
Job-years		13.4 (8.79)	11.1 (10.9)	0.20
Smoking status	Current	103 (65.2%)	29 (58%)	0.13
	Past	127 (80.4%)	34 (68%)	
	Never	31 (19.6%)	16 (32%)	
Number cigarettes per day		12.7 (11.9)	9.72 (10.3)	0.092
Pack-years		15.3 (15.7)	13.8 (17.5)	0.61
ETS exposure	Yes	140 (89.2%)	42 (84%)	0.54
	No	17 (10.8%)	8 (16%)	
ETS hours		5.32 (4.05)	3.52 (2.41)	<0.001*
% Leukocytes	Basophils	0.78 (1.29)	0.34 (0.82)	0.006*
	Eosinophils	0.84 (1.49)	1.22 (1.40)	0.11
	Monocytes	1.35 (1.47)	1 (1.68)	0.19
	Lymphocytes	42.1 (9.44)	41.2 (9.29)	0.60
	Neutrophils	53.8 (9.46)	55.3 (8.90)	0.32

Abbreviations: ETS, environmental tobacco smoke.

* *P*<0.05.

a Categorical variables are expressed as n (%), and continuous variables are expressed as mean (SD).

b P-values were obtained from Pearson’s chi-square test for categorical variables and Welch two-sample t-test for continuous variables and Wilcoxon rank-sum test for non-normally distribution variables.

All exposure indices were significantly higher in workers than controls (Table 2). Median air benzene levels for controls were not calculated as almost all of the controls, except for one, were exposed to benzene levels below the limit of detection (LOD). Both urinary SPMA and t,t-MA were significantly higher among the workers (all *P*<0.001). Median SPMA levels were approximately 15.5 µg/L among workers and 10.1 µg/L among controls, and median t,t-MA levels were 711 µg/L among workers and 25 µg/L among controls. The correlation between the three different exposure variables in workers was moderate: 0.23 between airborne log-benzene and SPMA (*P*<0.001); 0.40 between the two biomarkers SPMA and t,t-MA (*P*<0.001); and 0.48 between airborne log-benzene and t,t-MA (*P*<0.001). We found no statistically significant difference between workers and controls in the four methylation outcome variables as shown in Table 2. Because all of the controls except for one had air benzene below LOD, we did not use air benzene in the regression models reported below.

**Table 2:** A Comparison of exposure and methylation variables between workers and controls, Median (IQR).

Variables^a^	Workers (N = 158)						Controls (N = 50)						*P*-value^b^
	Min	p25	Median (IQR)	p75	Max	% <LOD	Min	p25	Median (IQR)	p75	Max	% <LOD	
Benzene, ppm	0.012^c^	0.19	0.46 (1.27)	1.47	23.9	2/158 (1.3%)	0.012^c^	0.012^c^	0.012 (0)^c^	0.012^c^	0.65	49/50 (98%)	−
SPMA, µg/L	0.24	9.95	15.5 (44.9)	54.9	349.4	0/158 (0%)	0.24	7.73	10.1 (6.6)	14.3	59.7	0/50 (0%)	<0.001*
t,t-MA, µg/L	25.0^d^	311.5	711 (1270)	1581	9961	13/158 (8.2%)	25.0^d^	25.0^d^	25.0 (75.0)^d^	100	754	33/50 (66%)	<0.001*
Alu, %	22.4	25.1	25.6 (1.03)	26.1	27.2	−	21.9	24.6	25.3 (1.6)	26.2	27.9	−	0.15
LINE-1, %	71.6	80.7	81.8 (1.97)	82.7	85.3	−	75.4	80.5	81.9 (2.23)	82.8	84.3	−	0.72
*MAGE,* %	47.0	90.4	91.6 (2.16)	92.5	96.4	−	86.2	90.2	91.6 (2.05)	92.3	93.7	−	0.60
*p15*, %	1.12	2.60	3.32 (1.86)	4.46	14.1	−	1.80	2.87	3.45 (1.23)	4.10	7.65	−	0.78

Abbreviations: SPMA, S-phenylmercapturic acid; t,t-MA, Trans-trans-muconic acid.

Data were missing and excluded in the analysis for the following variables: SPMA (10 individuals), t,t-MA (7 individuals), Alu (14 individuals), LINE-1 (15 individuals), *MAGE* (16 individuals) and *p*15 (28 individuals).

* *P*<0.05.

a All exposure and outcome variables are expressed as minimum (Min), 25^th^ quantile (p25), median (IQR), 75^th^ quantile (p75), maximum (Max).

b P-values were obtained from Wilcoxon’s rank sum test.

c Almost all controls (49/50) have values less than LOD of 0.023 ppm and were assigned a value corresponding to half of LOD in the analysis (i.e. 0.012 ppm).

d A majority of the controls (33/50) have values less than LOD of 50 µg/L and were assigned a value corresponding to half of LOD in the analysis (i.e. 25 µg/l).

Table 3 presents the OLS regression results for the association between percent methylation of each methylation variable with each urinary biomarker. The magnitudes of association estimates were expressed as % change in DNA methylation per an IQR increase in the exposure biomarkers. Using urinary SPMA as exposure, we found small but statistically significant decreases in the methylation levels of both LINE-1 (−0.089% per IQR increase, *P* = 0.035) and *p15* (−0.090% per IQR increase, *P = *0.048) with increasing SPMA in the unadjusted model. Only LINE-1 was significant when adjusted for all other covariates. LINE-1 methylation level was reduced by 0.14% (*P* = 0.009) with each IQR increase in urinary SPMA exposure after adjusting for age, sex, education, smoking and ETS hours. There were no significant associations with t,t-MA. Adjusting for the possible effects that may be due to inherent between-participant differences in relative proportions of the various leukocyte cell types (Table S4) did not alter the results, so heterogeneity in leukocyte cell type was not included in the final model.

**Table 3:** Association Estimates of Urinary Biomarkers, SPMA and t,t-MA, on Repeated-element and Gene-Specific Percent Methylation using OLS Regression.

		Unadjusted		Adjusted^a^			
Biomarker	Outcome	Coefficient^b^	*P-*value	Coefficient^b^	*P-*value	R^2c^	AIC
SPMA	Alu	0.010	0.72	0.0072	0.83	0.032	444.0
	LINE-1	−0.089	0.035*	−0.14	0.009*	0.054	581.1
	*MAGE*	0.025	0.80	0.011	0.93	−0.034	838.7
	*p15*	−0.090	0.048*	−0.077	0.20	0.021	562.2
t,t-MA	Alu	0.027	0.51	0.034	0.48	0.017	486.0
	LINE-1	−0.054	0.38	−0.044	0.56	0.012	633.0
	*MAGE*	0.056	0.71	0.034	0.85	−0.025	901.9
	*p15*	0.057	0.39	0.081	0.32	0.011	608.7

Abbreviations: SPMA, S-phenylmercapturic acid; t,t-MA, Trans-trans-muconic acid; AIC, Akaike’s information criterion.

* *P*<0.05.

a Model adjusted for age, sex, smoking history, education, ETS hours.

b Coefficient refers to the change in methylation % per IQR change in exposure variable.

c Adjusted R^2.^

**Figure 1:**
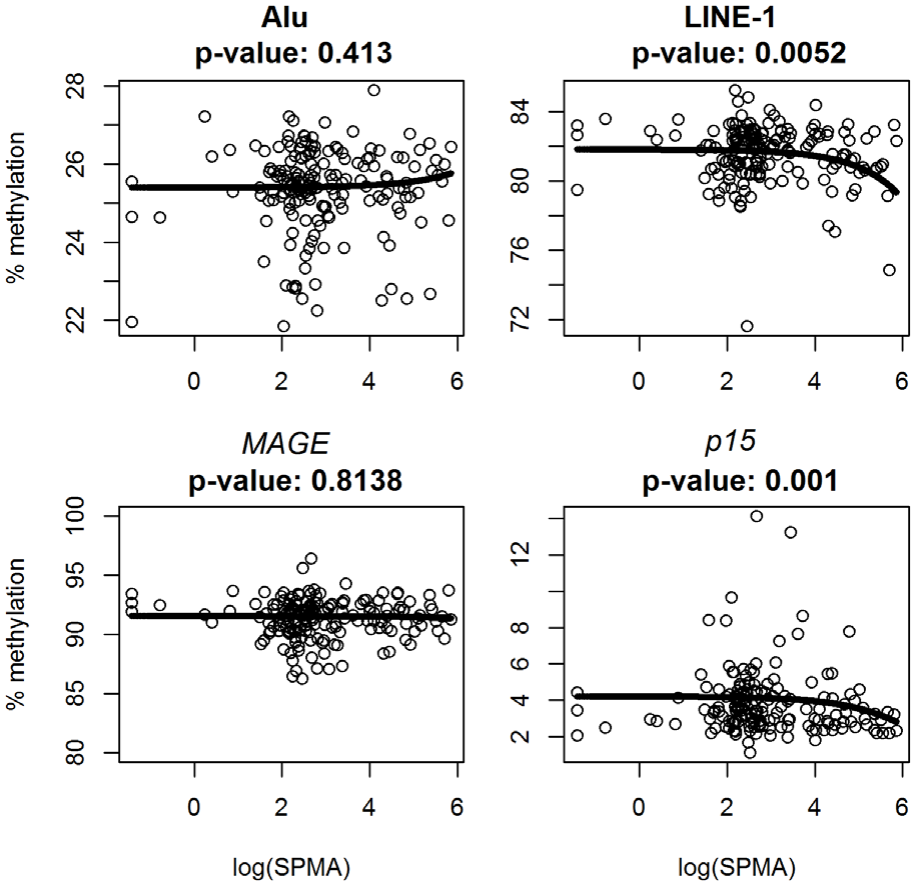
Association of S-phenylmercapturic acid (SPMA) with DNA methylation in Alu, LINE-1, *MAGE* and *p15* methylation. Fitted beta regression models of repeated-element and gene-specific methylation % versus log(SPMA), adjusted for potential confounders as described in the text. Lines correspond to fitted mean trajectories from beta regression models using the logit link, evaluated for hypothetical individuals with sample mean covariate values (petrochemical workers, non-smoking male, age: 40, ETS: 5.3 hours, education: middle-school). P-values shown correspond to main associations of SPMA in each model.

Table 4 shows the beta-regression results for the association between percent methylation of each methylation variable with each of the two urinary exposure variables. Using unadjusted beta-regression models, we found statistically significant but modest reductions in LINE-1 (0.085%, *P* = 0.033) and in *p15* (0.084%, *P* = 0.023) methylation levels with each IQR increase in urinary SPMA. After adjusting for all of the covariates, both LINE-1 and *p15* remained significant; we found a significant 0.15% decrease in LINE-1 (*P* = 0.005), and 0.096% decrease in *p15* (*P* = 0.001) methylation per an IQR increase in urinary SPMA. Significant associations were not observed with t,t-MA. Scatterplots showing SPMA and each of the methylation markers, as well as regression lines from beta-regression models are shown in Figure 1.

**Table 4:** Association Estimates of Urinary Biomarkers, SPMA and t,t-MA, on Repeated-element and Gene-Specific Percent Methylation using Beta-regression.

		Unadjusted		Adjusted^a^			
Biomarker	Outcome	Coefficient^b^	*P-*value	Coefficient^b^	*P-*value	R^2^ c	AIC
SPMA	Alu	0.010	0.71	0.023	0.41	0.050	−908.0
	LINE-1	−0.085	0.033*	−0.15	0.005*	0.10	−768.6
	*MAGE*	0.010	0.88	−0.010	0.81	0.000018	−652.4
	*p15*	−0.084	0.023*	−0.096	0.001*	0.070	−755.5
t,t-MA	Alu	0.028	0.51	0.013	0.70	0.048	−976.0
	LINE-1	−0.050	0.40	−0.0029	0.97	0.056	−834.4
	*MAGE*	0.018	0.86	−0.026	0.71	0.0021	−723.0
	*p15*	0.12	0.81	0.11	0.12	0.051	−824.2

Abbreviations: SPMA, S-phenylmercapturic acid; t,t-MA, Trans-trans-muconic acid; AIC, Akaike’s information criterion.

* *P*<0.05.

a Model adjusted for age, sex, smoking history, education, ETS hours.

b Coefficient refers to the change in methylation % per IQR change in exposure variable.

c Pseudo-adjusted R^2.^

In general, the results from beta-regression were overall consistent with those from OLS regression. However, beta-regression models had higher adjusted pseudo R^2^ values and lower AICs as compared to those generated by OLS regression models (Tables 3 and 4), indicating that beta regression performed better, in terms of model fit, than OLS regression models. We note that for the association of *MAGE* methylation with both SPMA and t,t-MA, OLS regression models showed negative adjusted R^2^ values, which indicate particularly inadequate fit to the methylation data [32], [33]. In addition, Pearson residual plots showed that beta regression generally provides better fit of the data (Figures S1 and S2).

## Discussion

In this study, we investigated the association between occupational benzene exposure with both repeated-element and gene-specific DNA methylation in a population of Bulgarian petrochemical workers. Using beta-regression, we found small but statistically significant associations of LINE-1 and *p15* hypomethylation with SPMA after adjusting for covariates. We also showed that beta-regression provided better goodness-of-fit, as reflected in higher adjusted pseudo R^2^ and lower AIC values, compared to standard linear OLS regression models. Our results show that exposure to benzene, as reflected in the internal dose measured by urinary SPMA was associated with both repeated-element and gene-specific hypomethylation.

Our finding showing a decrease in LINE-1 methylation with increasing urinary SPMA levels in beta-regression models is consistent with the global hypomethylation reported in response to various environmental exposures [17], [34]–[38] and has been suggested to represent an early step preceding the severe hypomethylation typical of leukemia and other malignancies [12], [39]–[42]. The observed association was small in size, consistent with previous results by Bollati et al. in a study of gas-station attendants and police officers exposed to low-level benzene [17]. Similar effect sizes on DNA methylation measured in blood leukocytes from healthy participants have been suggested to represent an early deviation from normal methylation patterns induced by benzene exposure [17].

We also found a decrease in *p15* methylation in relation to increasing SPMA levels, contrary to the hypermethylation of *p15* reported in the previous investigation by Bollati et al. [17]. Also, we did not find any significant associations with Alu and *MAGE* methylation, which showed a small but significant negative association with airborne benzene levels in the Bollati et al. study [17]. Several differences in the levels and sources of exposure might account for the discrepancies between the two studies. Bollati et al. studied traffic-exposed subjects in an urban area and investigated associations of DNA methylation with airborne benzene levels. In our study instead, we examined a population of industrial workers with higher workplace exposure levels, and evaluated associations with urinary biomarkers of benzene metabolism. The different settings of the exposures, as well as the different exposure levels may account for the discrepancies in the results.

This is the first study to compare the use of beta-regression and OLS regression methods for analyzing proportion methylation outcomes. We found that beta-regression provided a better goodness-of-fit, as reflected in higher adjusted pseudo R^2^ values and lower AIC values. This finding is consistent with the concept that beta-regression is more appropriate for analyzing proportion data, such as DNA methylation, compared to standard linear OLS regression models. We obtained negative adjusted R^2^ values for *MAGE* in the OLS regression model, because the adjusted R^2^ takes into account the number of parameters in the model by subtracting a function of that number from the value, thus potentially resulting in negative values in cases of particularly poor fit [32], [33]. Although results from beta-regression were overall consistent with OLS regression, in beta-regression we found some significant, but weak, associations of benzene exposure and DNA methylation which would have been missed by OLS regression. This finding suggests that beta-regression may be more sensitive for detecting small changes in percent methylation. This is due to the more flexible nature of the beta distribution, which unlike the Gaussian distribution having independent mean and variance parameters, allows for the mean and the variance to be related, where the variance is a function of the mean [43]. In fact, being a member of the exponential family, the beta distribution has a variance function which directly depends on the mean and, as such, differences in variance within a variable can be just as informative as differences in the mean [31]. In addition, beta-regression allows the use of various link functions linking the outcome measure to the set of explanatory covariates, allowing one to fit models with non-linear mean trajectories. Alternatively, simplex regression can also be used to fit data bounded in (0,1) and has been shown to give estimates concordant with beta regression [44].

In the present study, we found associations of urinary SPMA with DNA methylation markers, whereas no gross methylation differences were observed between exposed workers and controls. In general, biomarkers of exposure, including SPMA, are used to yield more accurate measures of biological dose than occupational group categories, as they reflect the level of absorption, metabolism, as well as of the clearance of the chemical. SPMA in particular, has been previously found to be associated with oxidative stress [21], a benzene-induced cellular stressor and well-established modifier of methylation patterns [45]. On the contrary, we did not observe any significant correlation of t,t-MA levels with DNA methylation. Previous studies have also found the correlation of both SPMA and t,t-MA with airborne benzene to be relatively low and SPMA to be a more reliable biomarker for benzene exposure due to its longer half-life than t,t-MA [46]–[48].

The present study has a number of limitations, including a relatively small number of participants; and possible unmeasured confounding, such as non-occupational benzene exposures (e.g. from vehicle traffic). It should also be noted that other factors that might affect DNA methylation, such as nutritional factors, were unmeasured and could not be adjusted for in this study. Our study has also several strengths including: accurate outcome assessment with pyrosequencing duplicates completed for each outcome; biomarkers measured to take into account the internal dose of each subject with complete data for urinary SPMA; and the use of a population of workers exposed to high benzene levels which represents a different setting than those investigated in previous epigenetic studies. The workers in our population were exposed to benzene for an average of 13.4 years, and therefore any potential aberrant changes in methylation if any, caused by chronic benzene exposure should have been clearly visible after such prolonged exposure to high levels of benzene. We also considered potential influences of various leukocyte cell types on between-subject heterogeneity in the response.

Our findings showed that occupational benzene exposure was associated with small but statistically significant DNA methylation changes in the workers. Our results lacked consistency across different biomarkers and exposure metrics and should therefore be interpreted with caution. Also, some of our findings, such as the negative associations between SPMA and DNA methylation of p15, are at variance with previous findings on benzene-exposed individuals [17]. Because DNA methylation is potentially modifiable through lifestyle and pharmacological interventions, if confirmed, our findings may open new paths for prevention against benzene-induced carcinogenesis. If benzene-related hypomethylation were to be found to be a mechanistic intermediate along the path linking benzene to its carcinogenic effects, then targeted interventions, such as providing benzene-exposed workers with dietary supplements, including folic acid which may contrast the association of benzene with DNA hypomethylation [49] could be developed and tested among benzene-exposed workers.

## Supporting Information

Figure S1 **OLS Pearson Residual Plots of the Association of S-phenylmercapturic acid (SPMA) with DNA methylation in Alu, LINE-1,** ***MAGE*** **and** ***p15*** **methylation.** OLS Pearson residuals of the association of S-phenylmercapturic acid (SPMA) with DNA methylation in Alu, LINE-1, *MAGE* and *p15* methylation %, adjusted for potential confounders as described in the text. Lines correspond to zero reference residual values on the y-axis.
(TIFF)

Figure S2 **Beta Regression Pearson Residual Plots of the Association of S-phenylmercapturic acid (SPMA) with DNA methylation in Alu, LINE-1,** ***MAGE*** **and** ***p15*** **methylation.** Beta Regression Pearson residuals of the association of S-phenylmercapturic acid (SPMA) with DNA methylation in Alu, LINE-1, *MAGE* and *p15* methylation %, adjusted for potential confounders as described in the text. Lines correspond to zero reference residual values on the y-axis.
(TIFF)

Table S1 **PCR Primers Concentrations and Cycling Conditions.**
(PDF)

Table S2 **Primers for PCR assay.**
(PDF)

Table S3 **Localization of Gene Promoters and Regions Amplified.**
(PDF)

Table S4 **Model Estimates of OLS and Beta Regression adjusting for Leukocyte Proportions.**
(PDF)
