# Supplementary figures and images for: Urinary Benzene Biomarkers and DNA Methylation in Bulgarian Petrochemical Workers: Study Findings and Comparison of Linear and Beta Regression Models
Source: PLoS One. 2012 Dec 5;7(12):e50471. doi: 10.1371/journal.pone.0050471 (PMC3515615; doi:10.1371/journal.pone.0050471)

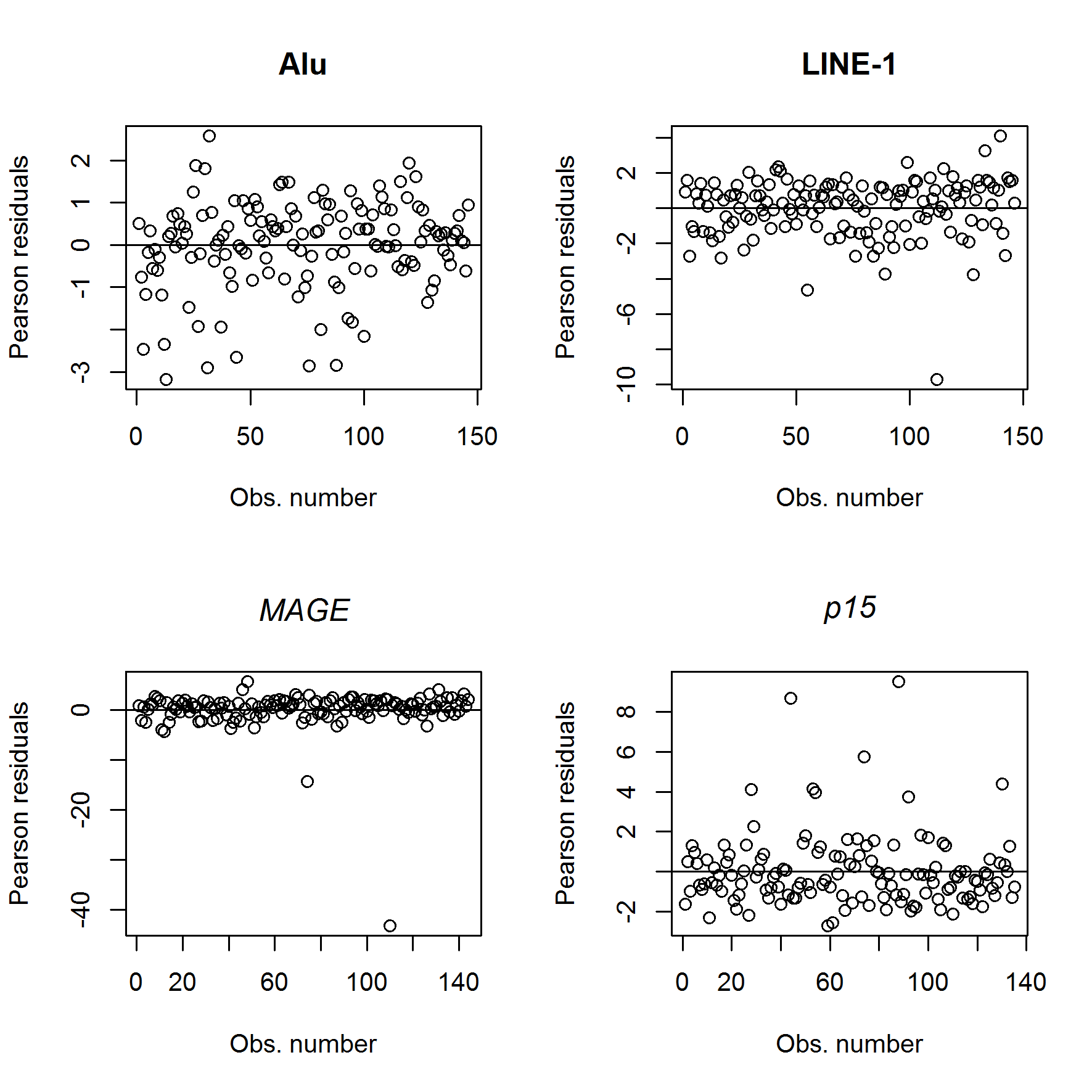

Supplement: Figure S1 — OLS Pearson Residual Plots of the Association of S-phenylmercapturic acid (SPMA) with DNA methylation in Alu, LINE-1, MAGE and p15 methylation. OLS Pearson residuals of the association of S-phenylmercapturic acid (SPMA) with DNA methylation in Alu, LINE-1, MAGE and p15 methylation %, adjusted for potential confounders as described in the text. Lines correspond to zero reference residual values on the y-axis. (TIFF) [file pone.0050471.s001.tifF]

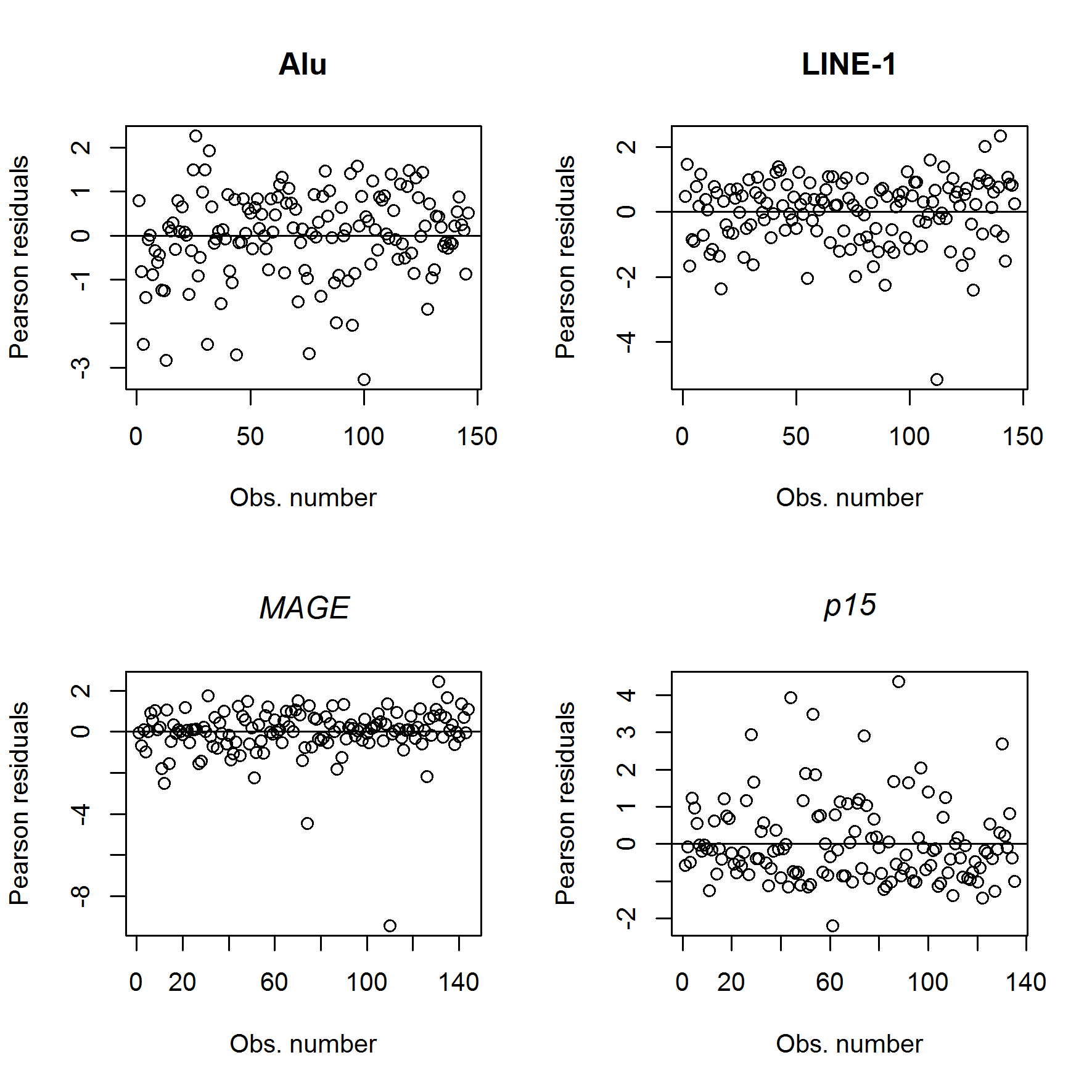

Supplement: Figure S2 — Beta Regression Pearson Residual Plots of the Association of S-phenylmercapturic acid (SPMA) with DNA methylation in Alu, LINE-1, MAGE and p15 methylation. Beta Regression Pearson residuals of the association of S-phenylmercapturic acid (SPMA) with DNA methylation in Alu, LINE-1, MAGE and p15 methylation %, adjusted for potential confounders as described in the text. Lines correspond to zero reference residual values on the y-axis. (TIFF) [file pone.0050471.s002.tifF]
