# Supplementary material for: Urinary Benzene Biomarkers and DNA Methylation in Bulgarian Petrochemical Workers: Study Findings and Comparison of Linear and Beta Regression Models
Source: PLoS One. 2012 Dec 5;7(12):e50471. doi: 10.1371/journal.pone.0050471 (PMC3515615; doi:10.1371/journal.pone.0050471)
Supplement: Table S1 — PCR Primers Concentrations and Cycling Conditions. (PDF) [file pone.0050471.s003.pdf]

Table S1. PCR Primers Concentrations and Cycling Conditions

	Alu	LINE	*MAGE*	*p15*
Primer Forward (10μM)	2μl	2μl	1μl	1μl
Primer Reverse (10μM)	2μl	2μl	1μl	1μl
Cycling Conditions	95°C for 90 sec, 43°C for 60 sec, 72°C for 120 sec for 45 cycles	95°C for 30 sec, 50°C for 30 sec, 72°C for 30 sec for 45 cycles	95°C for 30 sec, 55°C for 30 sec, 72°C for 30 sec for 45 cycles	95°C for 30 sec, 52°C for 30 sec, 72°C for 30 sec for 45 cycles
