# Supplementary material for: Urinary Benzene Biomarkers and DNA Methylation in Bulgarian Petrochemical Workers: Study Findings and Comparison of Linear and Beta Regression Models
Source: PLoS One. 2012 Dec 5;7(12):e50471. doi: 10.1371/journal.pone.0050471 (PMC3515615; doi:10.1371/journal.pone.0050471)
Supplement: Table S2 — Primers for PCR assay. (PDF) [file pone.0050471.s004.pdf]

Table S2. Primers for PCR assay

Sequence ID	Forward Primer (5' to 3')	Reverse Primer (5' to 3')	Sequencing Primer (5' to 3')	Sequence analyzed[a]
MAGE	Biotin - TATTGTGGGGTAGAGAGAAG	AAATCCTCAATCCTCCCTCAA	CCTCACAAAACCTAAATCAAATTCC	TTCA/GGACCA/GAACTCTACA/GGCCA/GTCCC
p15	GTTTTTTTTTAGAAGTAATTTAGG	Biotin - TCCTTCTACRACTTAAAACC	GTTAGGAAAAGTT	C/TGGAGTTAAC/TGATC/TGGTC/TGTTC/TGGTT
Alu	Biotin - TTTTTATTAAAAATATAAAAATT	CCCAAACTAAAATACAATAA	AATAACTAAAATTACAAAC	G/AC/TG/AC/TG/ACCACCA
LINE-1	TTTTGAGTTAGGTGTGGGATATA	Biotin - AAAATCAAAAAATTCCCTTTC	AGTTAGGTGTGGGATATAGT	TTC/TGTGGTGC/TGTC/TGTTTTTTAAGTC/TGGTTTG

[a] Nucleotides at which DNA methylation was measured are underlined.
