# Supplementary material for: Urinary Benzene Biomarkers and DNA Methylation in Bulgarian Petrochemical Workers: Study Findings and Comparison of Linear and Beta Regression Models
Source: PLoS One. 2012 Dec 5;7(12):e50471. doi: 10.1371/journal.pone.0050471 (PMC3515615; doi:10.1371/journal.pone.0050471)
Supplement: Table S3 — Localization of Gene Promoters and Regions Amplified. (PDF) [file pone.0050471.s005.pdf]

Table S3: Localization of Gene Promoters and Regions Amplified

Gene	Chromosome	Promoter		Amplicon		CpGs
		Start	End	Start	End	
MAGE	X	152485982	152486616	152486472	152486593	Pos1: 152486530
						Pos2: 152486526
						Pos3: 152486517
						Pos4: 152486514
p15	9	22008800	22009975	22009566	22009678	Pos1: 22009624
						Pos2: 22009633
						Pos3: 22009637
						Pos4: 22009641
						Pos5: 22009645
