# Supplementary material for: Urinary Benzene Biomarkers and DNA Methylation in Bulgarian Petrochemical Workers: Study Findings and Comparison of Linear and Beta Regression Models
Source: PLoS One. 2012 Dec 5;7(12):e50471. doi: 10.1371/journal.pone.0050471 (PMC3515615; doi:10.1371/journal.pone.0050471)
Supplement: Table S4 — Model Estimates of OLS and Beta Regression adjusting for Leukocyte Proportions. (PDF) [file pone.0050471.s006.pdf]

Table S4. Model Estimates of OLS and Beta Regression adjusting for Leukocyte Proportions

		OLS regression[a]				Beta-regression[a]			
		Exclude[b]		Include[c]		Exclude[b]		Include[c]	
	Outcome	Coef [d]	*P*-value	Coef [d]	*P*-value	Coef [d]	*P*-value	Coef [d]	*P*-value
SPMA									
	Alu	0.0072	0.83	0.011	0.76	0.023	0.41	0.032	0.13
	LINE-1	-0.14	0.009*	-0.14	0.01*	-0.15	0.005*	-0.11	0.04*
	MAGE	0.011	0.93	0.0047	0.97	-0.010	0.81	-0.012	0.78
	p15	-0.077	0.20	-0.095	0.80	-0.096	0.001*	-0.086	0.002*
t,t-MA									
	Alu	0.034	0.48	0.030	0.54	0.013	0.70	-0.009	0.76
	LINE-1	-0.044	0.56	-0.051	0.51	-0.0029	0.97	0.046	0.54
	MAGE	0.034	0.85	0.024	0.90	-0.026	0.71	0.010	0.89
	p15	0.081	0.32	0.081	0.35	0.11	0.12	0.12	0.06

* $P < 0.05$
[a] Model adjusted for age, sex, smoking history, education, ETS hours
[b] Model excluding percent leukocytes
[c] Model including percent leukocytes (basophils, eosinophils, lymphocytes, monocytes and neutrophils)
[d] Coefficient refers to the change in methylation % per IQR change in exposure variable
